# Identification of a Functionally Relevant Adeno-Associated Virus Rep68 Oligomeric Interface

**DOI:** 10.1128/JVI.00356-16

**Published:** 2016-07-11

**Authors:** Martino Bardelli, Francisco Zárate-Pérez, Leticia Agúndez, R. Michael Linden, Carlos R. Escalante, Els Henckaerts

**Affiliations:** a Department of Infectious Diseases, King's College London, London, United Kingdom; b Department of Physiology and Biophysics, Virginia Commonwealth University School of Medicine, Richmond, Virginia, USA; International Centre for Genetic Engineering and Biotechnology

## Abstract

The life cycle of the human parvovirus adeno-associated virus (AAV) is orchestrated by four Rep proteins. The large Rep proteins, Rep78 and Rep68, are remarkably multifunctional and display a range of biochemical activities, including DNA binding, nicking, and unwinding. Functionally, Rep78 and Rep68 are involved in transcriptional regulation, DNA replication, and genomic integration. Structurally, the Rep proteins share an AAA^+^ domain characteristic of superfamily 3 helicases, with the large Rep proteins additionally containing an N-terminal origin-binding domain (OBD) that specifically binds and nicks DNA. The combination of these domains, coupled with dynamic oligomerization properties, is the basis for the remarkable multifunctionality displayed by Rep68 and Rep78 during the AAV life cycle. In this report, we describe an oligomeric interface formed by Rep68 and demonstrate how disruption of this interface has drastic effects on both the oligomerization and functionality of the Rep proteins. Our results support a role for the four-helix bundle in the helicase domain of Rep68 as a bona fide oligomerization domain (OD). We have identified key residues in the OD that are critical for the stabilization of the Rep68-Rep68 interface; mutation of these key residues disrupts the enzymatic activities of Rep68, including DNA binding and nicking, and compromises viral DNA replication and transcriptional regulation of the viral promoters. Taken together, our data contribute to our understanding of the dynamic and substrate-responsive Rep78/68 oligomerization that is instrumental in the regulation of the DNA transitions that take place during the AAV life cycle.

**IMPORTANCE** The limited genome size of small viruses has driven the evolution of highly multifunctional proteins that integrate different domains and enzymatic activities within a single polypeptide. The Rep68 protein from adeno-associated virus (AAV) combines a DNA binding and endonuclease domain with a helicase-ATPase domain, which together support DNA replication, transcriptional regulation, and site-specific integration. The coordination of the enzymatic activities of Rep68 remains poorly understood; however, Rep68 oligomerization and Rep68-DNA interactions have been suggested to play a crucial role. We investigated the determinants of Rep68 oligomerization and identified a hydrophobic interface necessary for Rep68 activity during the AAV life cycle. Our results provide new insights into the molecular mechanisms underlying the regulation of the versatile Rep proteins. Efficient production of AAV-based gene therapy vectors requires optimal Rep expression levels, and studies such as the one presented here could contribute to further optimization of AAV production schemes.

## INTRODUCTION

Adeno-associated virus (AAV) is a human DNA virus of the family Parvoviridae with a unique dependence on helper viruses, such as adenovirus (Ad) or herpesvirus, for productive replication (1). In order to take advantage of host pathways and helper virus for productive replication with only a limited number of viral gene products at hand, AAV has evolved to combine multiple functions into single proteins. More specifically, a single open reading frame (ORF) generates the nonstructural Rep proteins that orchestrate the different aspects of the AAV life cycle, including transcriptional regulation, replication, packaging, and Rep-mediated integration. The four multidomain Rep proteins Rep40, Rep52, Rep68, and Rep78 are generated through the use of two promoters and alternative splicing (2, 3). All Rep isoforms share a superfamily 3 (SF3) helicase domain (HD) that combines ATPase and helicase activities (4, 5). The large Rep proteins, Rep68 and Rep78, further contain an N-terminal origin-binding domain (OBD) that specifically binds and nicks the AAV origin (5–7). Rep78 and Rep52 have an additional zinc finger domain that is involved in interactions with cellular proteins (8, 9). Differences in the domain composition of the Rep isoforms confer specific functionalities to the large and the small Rep proteins. Rep52 and Rep40 are necessary for efficient packaging of the viral DNA into preformed capsids but are dispensable for viral replication and integration (10). The DNA binding and nicking activities of the OBD of the large Rep proteins, on the other hand, are the basis for AAV DNA replication and integration (11, 12). More specifically, Rep78/68 bind to the Rep binding site (RBS) in the inverted terminal repeats (ITRs) and execute a site- and strand-specific nick at the nearby terminal resolution site (*trs*). This process is necessary for resolution of the ITRs and completion of the viral DNA replication cycle with the assistance of the host cell machinery (12, 13). Similarly, the DNA binding and endonuclease activities are required for mediation of integration at chromosomal target loci that contain RBS/*trs*, such as the integration hot spot *AAVS1* (14–16). In addition, efficient nicking of the *trs* at both the viral and cellular origins requires ATP-dependent helicase activity for the generation of an optimal single-stranded substrate (7, 17). Finally, both the OBD and the helicase domain have the ability to mediate the transcriptional regulation of viral and cellular promoters by two independent mechanisms conferring regulatory functions to both small and large Rep proteins (18–20).

Structurally, the Rep proteins belong to the SF3 of helicases, a group of multifunctional viral proteins combining a characteristic AAA^+^ motor domain, which couples ATP hydrolysis and DNA unwinding, with an origin-binding domain to achieve rapid origin melting (21). Other members of this family include the simian virus 40 (SV40) large T antigen (LTag) and papillomavirus (PV) E1. In contrast to the OBD of Rep, SV40 LTag and PV E1 lack endonuclease activity. The AAV large Rep proteins are also related to HUH endonucleases, which catalyze rolling-circle replication (RCR) in bacteriophages and geminiviruses, as well as in bacteria (22, 23), indicating a significant evolutionary conservation. The large AAV Rep proteins have a complex and dynamic oligomeric behavior that can adapt to the different DNA substrates present during the AAV life cycle, varying from the RBS-containing double-stranded DNA encountered during initial origin binding to the single-stranded DNA encountered after origin melting (24, 25). In contrast to the AAA^+^ domains of SV40 LTag and PV E1, which readily form hexameric rings (26, 27), Rep40 and Rep52 are monomeric, due to the absence of a complete oligomerization domain (OD) at the N terminus of the SF3 helicase domain (28). Mutation of the corresponding OD in SV40 LTag and PV E1 has been shown to prevent their oligomerization and to disrupt the replication of SV40 and PV, respectively (29, 30). Intriguingly, a similar OD was also found in oligomeric HUH endonucleases, such as RepB from the pMV158 streptococcal RCR plasmid, despite the absence of a helicase domain (31). In the large Rep proteins, the linker connecting the OBD and the helicase domain provides the residues necessary to complete the OD and plays a crucial role in the oligomerization of the large Rep proteins (28, 32). Thus, the OBD, linker, and helicase domain effectively interact cooperatively to promote oligomerization.

While recent findings have significantly contributed to the understanding of the determinants of Rep oligomerization (25, 28, 32), its relevance in the context of the AAV life cycle remains to be elucidated. In order to gain an understanding of how oligomerization contributes to the multiple enzymatic functions of the Rep proteins, we took advantage of our previous findings, which showed that the linker domain and, in particular, the N-terminal linker residue Y224 are essential for Rep oligomerization (28). This residue was found in a position equivalent to that of the residues in SV40 LTag and PV E1 known to be crucial for the formation and maintenance of the oligomeric interface (28). Here we describe an oligomeric interface for Rep, identified in a dimeric complex modeled using the Rep40 structure with a predicted extended N-terminal α-helix to complete the OD (33). This model highlights a potential role for Y224 and I251 in the formation of the oligomeric interface; site-directed mutagenesis confirmed that oligomerization is indeed hampered when Y224 and I251 are altered. Moreover, we could demonstrate that mutations that lead to a disruption of the interface result in defects in DNA binding and *trs* nicking and alter the expression levels of the viral proteins, with severe consequences on viral DNA replication and production of infectious virus.

## MATERIALS AND METHODS

### Protein production and purification.

All mutations were generated in the pHisRep68/15b plasmid, which contains the AAV serotype 2 (AAV2) Rep68 ORF subcloned in the vector pET-15b (Novagen), using a QuikChange mutagenesis kit (Agilent Technologies Inc.). All proteins were expressed in Escherichia coli BL21(DE3) cells (Novagen) and purified as described previously (28). In brief, cell pellets were lysed in Ni-buffer A (20 mM Tris-HCl [pH 7.9 at 4°C], 500 mM NaCl, 5 mM imidazole, 10% glycerol, 0.2% CHAPS {3-[(3-cholamidopropyl)-dimethylammonio]-1-propanesulfonate}, 1 mM TCEP [Tris(2-carboxyethyl)phosphine hydrochloride]) and purified using an Ni column. The hexahistidine tag was removed using PreScission protease, and Rep68 was further purified by gel filtration chromatography using a HiLoad Superdex 200 16/60 column (GE Healthcare) and size exclusion buffer (25 mM Tris-HCl [pH 8.0], 200 mM NaCl, 2 mM TCEP). Rep68 wild-type (WT) and mutant proteins were concentrated to 10 mg/ml, flash-frozen in liquid N_2_, and kept at −80°C.

### Sedimentation velocity experiments.

Analytical ultracentrifugation experiments were carried out using a Beckman Optima XL-I analytical ultracentrifuge (Beckman Coulter Inc.) equipped with both four- and eight-position rotors. Protein samples (420 μl; final concentration, 10 μM) were loaded in the cells, and in all cases, buffer containing 25 mM Tris-HCl, pH 8.0, and 200 mM NaCl was used. Samples were centrifuged in 2-sector carbon-filled Epon centerpieces at 25,000 rpm at 20°C. At least 200 scans were collected at 5-min intervals at 25,000 rpm. Sedimentation velocity-concentration profiles were collected using both UV absorption (280 nm) and Rayleigh interference scanning optics. Results were analyzed using the SEDFIT program (34, 35).

### AAV infectious particle assay.

293T cells were triple transfected with an AAV2 inverted terminal repeat (ITR)-containing plasmid carrying a CAG-controlled green fluorescent protein (GFP) gene (pTRUF11), a helper plasmid expressing AAV2 Rep (WT or mutants) and Cap, and a third construct containing the adenovirus helper functions (HGTI plasmid) (36, 37). The mutations in Rep were confirmed in all plasmids by sequencing (Eurofins). After 72 h, the supernatant was harvested and spun to clear the cellular debris, and increasing volumes of supernatant were used to transduce HeLa cells. The percentage of GFP-positive HeLa cells was determined at 48 h postransduction by flow cytometry (FACSCanto; BD Biosciences).

### qPCR-based replication assay.

293T cells were transfected with polyethylenimine (Polysciences, Inc.) and the infectious AAV plasmid pAV2 (38) or its mutant versions and superinfected 4 h later with adenovirus serotype 5 (Ad5) at a multiplicity of infection (MOI) of 5. After 72 h, cells were harvested in phosphate-buffered saline (PBS) and pelleted, and the pellet was divided into 4. One-fourth was used for RNA extraction, one-fourth was used for protein extraction, one-fourth was used for total DNA extraction, and the last quarter was used for Hirt extraction of low-molecular-weight DNA.

Total DNA was extracted using a Qiagen DNeasy blood and tissue DNA extraction kit. Viral DNA was quantified by real-time PCR using the SYBR green JumpStart *Taq* ReadyMix for quantitative PCR (qPCR; Sigma-Aldrich) and an ABI Prism system (Applied Biosystems). Cap primers (forward [fw] primer, TTCTCAGATGCTGCGTACCGGAAA; reverse [rv] primer, TCTGCCATTGAGGTGGTACTTGGT) and a pAV2-based standard curve were used for absolute quantification; the signal was normalized to that of cyclophilin (fw primer, TGCTGGACCCAACACAAATG; rv primer, TGCCATCCAACCACTCAGTCT).

### Analysis of replicative intermediates.

293T cells were treated as described above for the qPCR-based replication assay. Low-molecular-weight DNA was extracted using a modified version of the Hirt extract procedure (39). Briefly, cells were lysed in Hirt lysis buffer (0.6% SDS, 10 mM Tris, pH 7.5, 10 mM EDTA) and treated with proteinase K (Thermo Fisher) to digest proteins. The high-molecular-weight DNA was precipitated and discarded. The low-molecular-weight DNA was then purified by phenol extraction, followed by sodium acetate and isopropanol precipitation. The precipitated DNA was washed and resuspended in DNase-free water. The extracts were digested with the restriction enzyme DpnI (New England BioLabs) to digest input DNA. Samples were run on a 0.8% agarose gel at 30 V overnight and transferred to a nitrocellulose membrane by the Southern blotting method. The membranes were hybridized overnight in 0.75 nylon wash buffer (40.6 g Na_2_HPO_4_, 18.65 g EDTA, and 500 g SDS in 3.58 liters of double-distilled H_2_O, pH 7.2) at 65°C with a radiolabeled Rep probe (fw primer, 5′-AACTGGACCAATGAGAACTTTCC-3′; rv primer, 5′-A AAAAGTCTTTGACTTCCTGCTT-3′) or an ampicillin probe (fw primer, 5′-AATCAGTGAGGCACCTATCTCAGC-3′; rv primer, 5′-AACTCGGTCGCCGCATACACTATT-3′) to control for DpnI digestion. The probes were labeled with a Prime-It RmT random primer labeling kit from Stratagene and [^32^P]dCTP (PerkinElmer). The membranes were exposed to a PhosphorImager screen overnight. Images were acquired using a Typhoon PhosphorImager (Molecular Dynamics) and analyzed with ImageQuant TL software (GE Healthcare Life Sciences).

### Fluorescence anisotropy DNA binding assay.

Binding assays were performed using a fluorescein-labeled 41-mer containing *AAVS1* or p5 RBS sequences. The sequences used were 5′-TGGCGGCGGTTGGGGCTCGGC*GCTCGCTCGCTC*GCTGGGCG-3′ (*AAVS1*) and 5′-ACCGGGCAAAATGGAGACCCTGCGTGCTCACTCGGGCTTAA-3′ (p5), where the *AAVS1* and p5 sequences are in italics (40, 41). Rep68 WT and mutant proteins at concentrations ranging from 5 nM to 3 μM were mixed with DNA (5 nM) in a final volume of 300 μl using the following buffer: 25 mM HEPES (pH 7.0), 100 mM NaCl, 1 mM TCEP. Fluorescence readings were taken on a PC1 fluorimeter (ISS, Inc.) with excitation and emission filters at 490 and 520 nm, respectively. The tubes were equilibrated at 20°C for 20 min before measurement. Each anisotropy point is the average of 10 measurements. Anisotropy is calculated as the ratio of the difference between the vertical and horizontal emission intensities to the total normalized intensity. The fraction of DNA bound (*B*) was calculated using the following equation: *B* = ([*A*]_*x*_ − [*A*]_DNA_)/([*A*]_final_ – [*A*]_DNA_), where [*A*]_*x*_ represents the anisotropy measured at protein concentration *x*, [*A*]_DNA_ is the anisotropy of free fluorescent DNA, and [*A*]_final_ is the anisotropy at saturation. The data were fit to a single binding site model by use of the Hill coefficient and the program Origin (Origin Labs). Each experiment was done in triplicate.

### Helicase assay.

The substrate used in the helicase assay is a heteroduplex DNA consisting of an 18-bp duplex region with a 10-nucleotide 3′ tail at the bottom strand, referred to as 18ADT10A. The top strand (trap DNA) is labeled at the 5′ end with fluorescein and is released upon unwinding. The sequences used were 5′-F-CATATGGAGCAGAACAGA-3′ for the trap DNA and 5′-AGACAAGACGAGGTATACAAAAAAAAAA-3′ for the complementary strand.

All reactions were performed in a buffer containing 25 mM HEPES, 50 mM NaCl (pH 7.0) at a total volume of 50 μl. Protein (1 mM) was mixed with 0.5 mM double-stranded fluorescein-labeled DNA (18ADT10A) and 2.5 μM single-stranded DNA (18-nucleotide sense DNA), and the mixture was then added to the mix of buffer described above containing 5 mM ATP and 5 mM MgCl_2_. The reaction mixture was incubated at 25°C for 1 h. EDTA was used at a final concentration of 20 μM to stop the reaction. Aliquots of 10 μl were loaded in a 12% bisacrylamide gel (30%) (19:1) using 6× loading dye (0.25% xylene cyanol FF, 30% glycerol). For the densitometry and analysis of the bands, a Gel Doc EZ imager was used, together with the automatic lane and band detection tool. Lane background subtraction, white illumination, and an activation time of 300 s were used for the analysis.

### scDNA nicking assay.

Supercoiled DNA (scDNA) nicking activity for Rep68 was assayed as described previously (14). Briefly, assays were performed in 30-μl reaction mixtures containing 30 mM HEPES-KOH (pH 7.5), 7 mM MgCl_2_, 0.5 mM dithiothreitol, 4 mM ATP, 40 mM creatine phosphate (Sigma), and 1 μg creatine phosphokinase (Sigma) in 15 mM (final concentration) NaCl. One hundred nanograms of supercoiled plasmid DNA and 200 ng of purified His-Rep68 (or mutants) were added to the reaction mixtures. All samples were incubated for 1 h at 37°C; the reaction was terminated by adding 10 μl of stop reaction mixture (proteinase K [1.2 μg/μl], 0.5% SDS, 30 mM EDTA, pH 7.5) and incubating for 1 h at 37°C. Samples were resolved in a 1% agarose gel (1× Tris-acetate-EDTA [TAE]), which was subsequently stained with ethidium bromide (0.3 μg/ml) in 1× TAE. The plasmids carrying scDNA used in this assay were pRVK (which contains *AAVS1* from nucleotides 1 to 3536) and a mutated version containing a mutant *trs* sequence (42).

### Western blotting.

Proteins were extracted from cells transfected and infected as described above for the replication assays. Cells were lysed in radioimmunoprecipitation assay buffer, and the cleared lysate was run on a 12% acrylamide gel. The proteins were transferred onto a nitrocellulose membrane (GE Healthcare) and immunoblotted using anti-Rep antibody (1/100 dilution; clone 303.9; Progen), anti-Cap antibody (1/500 dilution; clone B1; American Research Products), and anti-HSP90 antibody (polyclonal, 1/5,000 dilution; Santa Cruz). All antibodies were incubated in blocking buffer (5% nonfat dried milk in PBS containing 0.1% Tween 20). Images were acquired and analyzed using an ImageQuant apparatus (GE Healthcare).

### Real-time quantitative RT-PCR.

293T cells were transfected and infected as described above for the replication assays. Total RNA was extracted using an RNeasy kit (Qiagen) after DNase I (Qiagen) treatment for 15 min at 37°C. Reverse transcription (RT) was performed using a high-capacity reverse transcription kit (Applied Biosystems). cDNA was quantified by real-time qPCR on an ABI Prism system (Applied Biosystems) using the TaqMan Universal PCR master mix (Life Technologies) and custom-designed primer-probe mixes (Eurofins). The following primers were used: p5 fw (5′-AACAAGGTGGTGGATGAGT-3′), p5 rv (5′-CGTTTACGCTCCGTGAGATT-3′), p19 fw (5′-TCACCAAGCAGGAAGTCAAAG-3′), p19 rv (5′-CCCGTTTGGGCTCACTTATATC-3′), p40 fw (5′-GGAAGCAAGGCTCAGAGAAA-3′), and p40 rv (5′-CCTCTCTGGAGGTTGGTAGATA-3′). The following probes were used: p5 (5′-FAM-ACGTGGTTGAGGTGGAGCATGAAT-TAM-3′), p19 (5′-FAM-ACGTGGTTGAGGTGGAGCATGAA-TAM-3′), and p40 (5′-FAM-AGGAAATCAGGACAACCAATCCCGT-TAM-3′), where FAM is 6-carboxyfluorescein and TAM is 6-carboxytetramethylrhodamine. Relative expression levels were determined by the ΔΔ*C_T_* threshold cycle (*C_T_*) quantification method (43), using 18S rRNA (TaqMan predeveloped assay reagents, human 18S rRNA; Applied Biosystems) as a housekeeping reference gene.

## RESULTS

### Y224 forms hydrophobic interactions necessary for Rep68 oligomerization.

**FIG 1:**
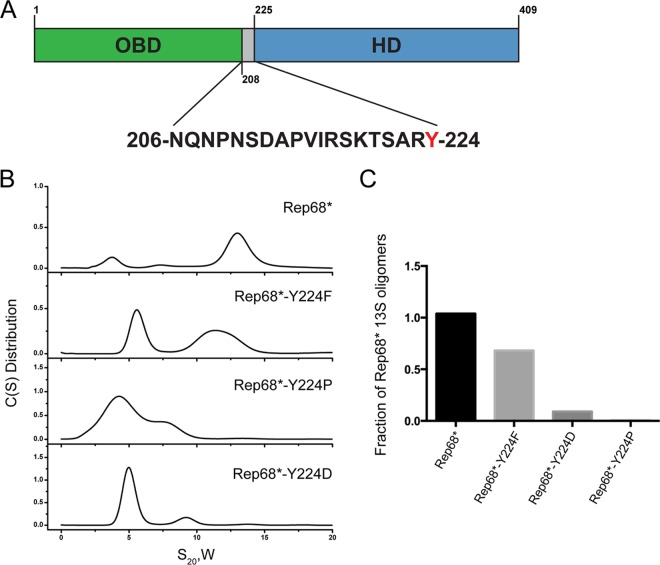
Role of Tyr224 in Rep68 oligomerization. (A) Schematic diagram of Rep68. The sequence of the linker is shown, and Y224 is highlighted in red. (B) Sedimentation velocity analysis of Rep68 and different Y224 mutants. C(S), sedimentation coefficient distribution. (C) Quantification of the amount of 13S species formed by Y224 mutants. Rep68* refers to the C151S mutant, which is functionally equivalent to WT Rep68 but prevents protein aggregation in solution (25). All mutant proteins were generated in the context of Rep68*.

Previous studies using Rep68* (or Rep* for short), a C151S Rep mutant that is functionally equivalent to WT Rep68 but that prevents protein aggregation in solution (25), showed that Rep68 exists as a mixture of oligomers in solution. More specifically, two major populations have been observed by sedimentation velocity experiments, including a monomer-dimer peak that sediments at ∼3S and oligomeric rings that sediment at 13S (25). We also showed that replacement of the tyrosine positioned at the C-terminal end of the linker in Rep68 by the smaller residue alanine disrupts its oligomerization; this is presumably because of a reduction in the surface-exposed area (Fig. 1A) (28). To further confirm this hypothesis, we mutated the tyrosine to phenylalanine (Phe), proline (Pro), or aspartic acid (Asp) and performed sedimentation velocity experiments to study how these mutations affect oligomerization. Figure 1B shows that replacement of the tyrosine with the small-side-chain amino acids Pro and Asp had a drastic effect on the sedimentation profile of Rep68. The 13S peak disappeared, and the most prominent population present in solution had a sedimentation coefficient of about 5S, suggestive of low-molecular-weight oligomers (Fig. 1B). Exchanging the tyrosine with the bulky aromatic Phe resulted in the appearance of two peaks, one with a sedimentation coefficient of 5S, similar to what we observed for the other mutants, and a second peak of about 12S, which was indicative of the formation of larger oligomers. This 12S population, however, had a sedimentation coefficient smaller than what we observed with Rep68, potentially suggesting that the Y224F mutant forms different oligomeric species. Figure 1C shows the quantification of the 13S population that was formed in the presence of the different mutations. Taken together, these results demonstrate that the bulky aromatic character of the Y224 residue is pivotal for Rep68 oligomerization and suggest that Y224 may participate in hydrophobic interactions as part of an oligomeric interface.

### Generation of a Rep68 oligomeric interface model.

**FIG 2:**
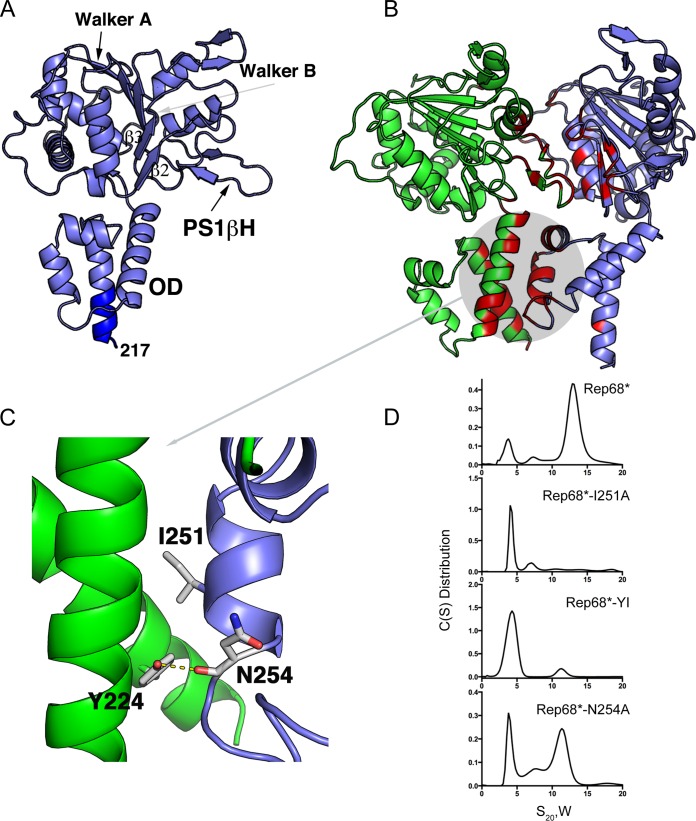
Model of a Rep-Rep interface. (A) Ribbon representation of Rep40 extended to residue 217 as an α-helix highlighted in dark blue. The Walker A and Walker B motifs, PS1βH, the β2β3 loop, and OD are indicated. (B) Model of a dimeric Rep complex. The structures participating in the interface are highlighted in red and magenta. (C) Close-up of the interactions formed by residue Y224, including the hydrophobic interaction with I251 and the hydrogen bond with the backbone of N254. (D) Sedimentation velocity analysis of the Rep68 I251A, Y224A-I251A (YI), and N254A mutants. All proteins were analyzed at a concentration of 1 mg/ml, as described in Materials and Methods.

To further determine whether Y224 participates directly in forming an oligomeric interface, we modeled an oligomeric Rep dimer using the available structure of Rep40 (PDB accession number 1S9H), which spans residues 225 to 490 of Rep68 (33). We added the interdomain linker residues 217 to 224 to the known Rep40 structure as an extended α-helix on the basis of secondary structure predictions (28), resulting in a Rep molecule containing residues 217 to 490 (Fig. 2A). Two of the three molecules found in the asymmetric unit of Rep40 crystals formed a pseudodimer. The interface formed in this dimer was similar to the oligomeric interface described for other SF3 helicase structures but is not optimal to perform catalysis (44). We used this dimer with the addition of the linker residues as our initial interface model, and we refined it by carrying out rigid body and side chain conformation optimization using the RosettaDock server (45, 46). Strikingly, the top 10 models generated had almost identical interfaces, as analyzed by the program PISA (47), suggesting that our model was robust. A representation of the Rep interface model is shown in Fig. 2B. The interface buries a total of 1,992 Å of solvent-accessible area and includes residues from all the helices in the oligomerization domain (OD), the presensor 1 β-hairpin (PS1βH), the β2β3 loop, and residues from the Walker A and Walker B motifs (Fig. 2B). A closer analysis revealed that the modeled linker residues participate in the interface. In particular, the conserved aromatic residue Y224, which is at the end of the linker region, is an important component of the oligomeric interface. In agreement with the results shown in Fig. 1, it participates in the formation of a hydrophobic pocket. Among the residues from the neighboring subunit interacting with Y224, residue I251, at a distance of 3.6 Å, takes part in a hydrophobic interaction, whereas N254 contributes to a hydrogen bond via its main chain carbonyl oxygen (Fig. 2C).

### Mutations leading to disruption of the interface affect oligomerization of Rep68.

Based on these observations, we generated a Rep68* mutant with the I251A substitution (Rep68*-I251A mutant) and we assessed the consequences of mutating this residue alone or in combination with Y224 on Rep68* oligomerization. Figure 2D shows sedimentation velocity profiles illustrating that these mutants are mostly monomeric, thus validating the prediction from our model. A single N254A mutant was also evaluated to confirm that the side chain of this residue does not contribute to the oligomeric interface. As predicted, because N254 forms a hydrogen bond through its main chain carbonyl oxygen rather than participates directly in the hydrophobic interface, the N254A mutation showed only a mild effect on sedimentation. Furthermore, the oligomerization defects which we observed when testing the different Y224 mutants (Fig. 1C) could also be explained by our modeled interface: smaller hydrophobic residues increase the solvent-accessible area and destabilize the interface, while a larger bulky residue maintains the hydrophobic pocket and possibly affects only the formation of the hydrogen bond between Y224 and N254, thus causing a milder defect. Finally, the Y224P mutation had the strongest effect, as it probably disrupts the helical character of this region and may affect the overall OD structure. To investigate whether the interface that we identified is biologically relevant and thus involved in Rep functions, we assessed how the observed disruptions in the oligomerization profile of Rep68 affect the AAV life cycle.

### Rep68 oligomerization is necessary to support the AAV life cycle.

**FIG 3:**
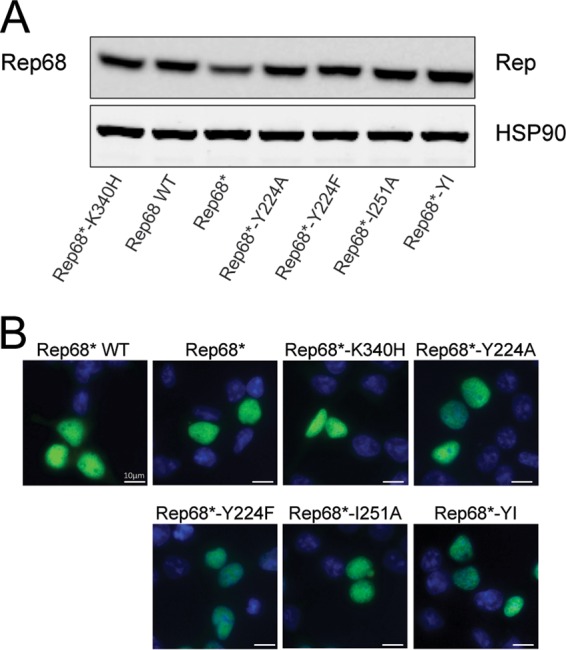
The interface mutants are stable and localize to the nucleus. (A) WT Rep68 or Rep68 mutants were transfected in 293T cells and tested for expression levels by Western blotting at 36 h posttransfection. Anti-HSP90 antibody was used as loading control to ensure that a similar amount of protein was used under each condition. (B) 293T cells transfected with WT Rep68 or Rep68 mutants were assessed at 48 h posttransfection by immunostaining. Merged images showing green (Rep) and blue (DAPI [4′,6-diamidino-2-phenylindole]-stained nuclei) channels are presented. Rep68*-K340H was used as a control for nuclear localization of a well-characterized nonfunctional Rep mutant.

First, we verified if the aforementioned mutant Rep68 proteins were stable and localized correctly to the nucleus, where they support AAV replication. We transfected 293T cells with constructs expressing Rep68 or the oligomerization mutants under the control of the cytomegalovirus promoter and assessed Rep68 protein stability and localization at 8 h posttransfection. Figure 3 demonstrates that all the mutants were expressed at levels comparable to those observed for WT Rep68 and translocated to the nucleus, as expected.

**FIG 4:**
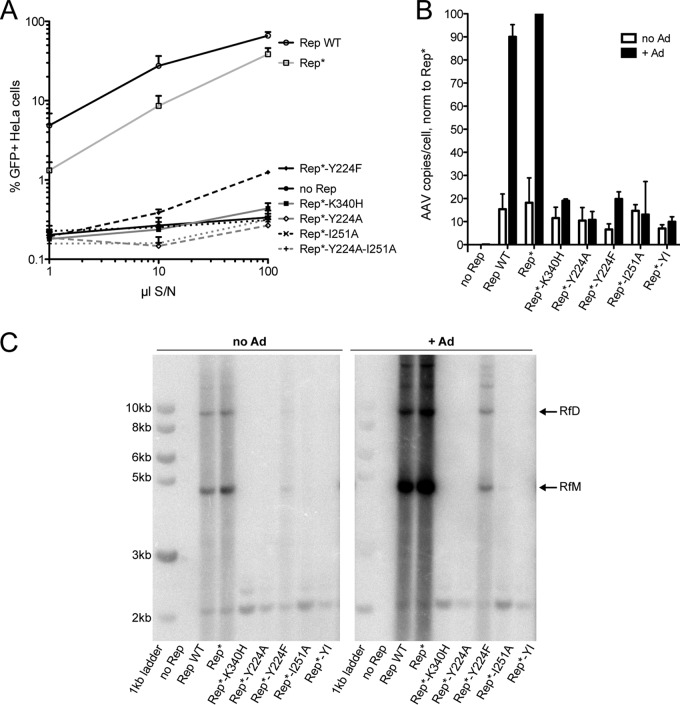
Interface mutants do not support the AAV life cycle. (A) Increasing volumes of supernatant from 293T cells producing recombinant AAV-GFP in the presence of WT Rep68 or Rep68 mutants were used to infect HeLa cells, and the percentage of GFP-positive (GFP^+^) cells was determined by fluorescence-activated cell sorting analysis. S/N, supernatant. Data are from three independent experiments and are represented as the mean ± SEM. (B) AAV DNA replication under permissive (+Ad) and nonpermissive (no Ad) conditions was quantified by quantitative PCR. Data from three experiments were normalized (norm) to those obtained under permissive (Rep*+Ad) conditions and are represented as the mean ± SEM. (C) AAV replicative intermediates generated under the same conditions shown in panel B were visualized by Southern blotting using a Rep-specific probe. RfM, monomeric replicative form; RfD, dimeric replicative form.

Next, we assessed how disruptions in the oligomerization profile of Rep68 affect the AAV life cycle. We first compared the ability of the Rep oligomerization mutants to produce infectious AAV particles to that of WT Rep68 and Rep68* (Fig. 4A). As a negative control, we used the nucleoside triphosphate-binding mutant K340H, which is deficient in ATPase and helicase activity and does not support AAV replication (48, 49). The K340H mutant, however, was still able to oligomerize and has been shown to have a dominant negative phenotype (48–50). Recombinant AAV2-GFP was produced in 293T cells by transfection of an ITR-containing plasmid carrying a GFP expression cassette together with plasmids encoding the adenovirus helper functions, AAV2 Cap, and WT Rep68, Rep68*, or the interface mutants. Increasing volumes of supernatant collected from the cultures of AAV-producing cells were added to HeLa cells in order to assess the infectivity of the produced virus. Figure 4A shows that the Y224A Rep mutant did not support the production of infectious AAV particles, as was previously reported by us (28). The mutant with the more conservative mutation, Y224F, which retained the potential to partially oligomerize (Fig. 1C), was severely impaired but was not entirely deficient in producing infectious AAV. Mutating I251 to alanine on the opposite side of the predicted interface, however, reproduced the phenotype observed with the Y224A mutant. Not surprisingly, the Y224A-I251A double mutant also failed to produce infectious AAV particles (Fig. 4A).

To evaluate if the failure to produce infectious particles was due to a defect in AAV DNA replication, we determined the number of AAV genomes in the 293T producer cells by qPCR (Fig. 4B) and studied the replicative intermediates formed during AAV replication by Southern blotting (Fig. 4C). Both assays confirmed that the Y224A and I251A interface mutants and the Y224A-I251A double mutant all failed to support AAV DNA replication. Similarly to what we observed in the infectious particle production assay, the Y224F mutant supported AAV replication but did so at levels significantly lower than those observed for Rep WT or Rep*. In addition, Fig. 4C shows that replication in the presence of the Y224F mutant resulted in the formation of the expected replicative intermediates. Background replication could be observed in the absence of adenovirus due to the presence of E1A and E1B in 293T cells (51). Altogether, these results suggest that the oligomerization interface mutants fail to sustain AAV DNA replication and therefore cannot support the production of infectious AAV particles.

### Rep68 oligomerization mutants are deficient in RBS-specific DNA binding and site- and strand-specific nicking but maintain the ability to unwind unspecific DNA substrates.

In order to determine the cause of the replication defect of the oligomerization-deficient mutants, we assessed various biochemical activities *in vitro*. Rep has three well-characterized enzymatic functions—RBS-specific DNA binding, *trs* nicking, and ATP-dependent DNA unwinding—all of which are necessary for AAV DNA replication and targeted genome integration. Table 1 shows the binding constants of Rep68* and mutant Rep68* proteins on p5 and *AAVS1* RBS-containing double-stranded DNA substrates. As expected, both Rep68* and the control, Rep68*-K340H, efficiently bound the specific DNA substrates (25, 52). The mutant that retained some residual replication potential, Rep68*-Y224F, also efficiently bound the p5 and *AAVS1* DNA substrates. All other mutants, however, lost the ability to bind both DNA substrates, with the exception of Rep68*-I251, which maintained its ability to bind *AAVS1*-containing DNA, albeit with a 10-fold lower affinity than its WT counterpart. These results suggest that some level of oligomerization is necessary for efficient RBS-specific DNA binding by Rep68 and that the oligomeric properties of the mutant Rep68*-Y224F are sufficient for DNA binding.

**TABLE 1:** Binding constants of Rep68* and interface mutants on *AAVS1* and p5 RBS-containing DNA

Protein	Binding constant (nM)	
	*AAVS1*-41	p5-41
Rep68*	128	203
Rep68*-K340H	123	136
Rep68*-Y224A	ND^*a*^	ND
Rep68*-Y224F	221	311
Rep68*-I251A	1,438	ND
Rep68*-YI	ND	ND

a ND, not determined due to poor binding.

**FIG 5:**
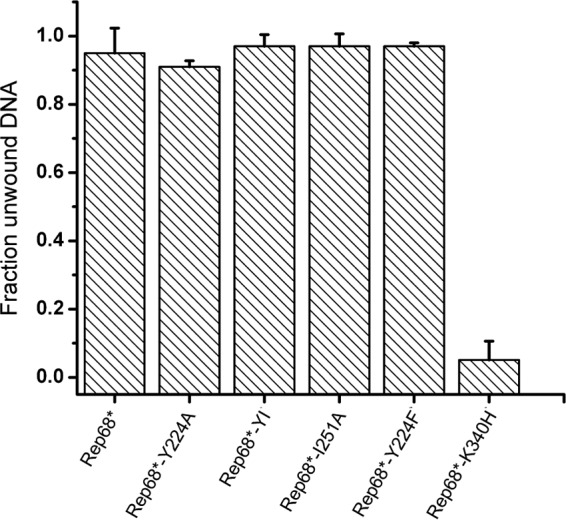
Comparison of the helicase activity of the interface mutants. The ability of the Rep68 interface mutants to unwind a fluorescein-labeled heteroduplex DNA substrate was assayed. Data are from three independent experiments and are represented as the mean ± SEM.

**FIG 6:**
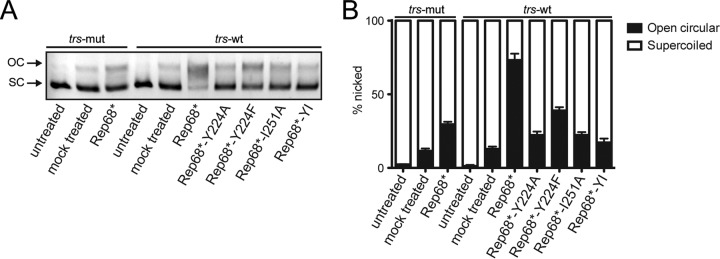
Rep-mediated nicking of supercoiled plasmid. Supercoiled (SC) plasmid DNA containing an RBS and a *trs* was mixed with Rep68* or the interface mutants. If the endonuclease activity is intact, Rep nicks and relaxes the plasmid conformation to an open circular (OC) form, which can be readily distinguished by agarose gel electrophoresis. A *trs* mutant (*trs*-mut) plasmid that was not nicked by Rep was used as a control. Untreated DNA was left untouched, while mock treated plasmid was incubated in reaction buffer for 1 h at 37°C in the absence of protein. (A) Representative agarose gel electrophoresis image. (B) Quantification of nicking from four independent nicking experiments. Data are represented as the mean ± SEM.

To test the ability of the Rep68 mutants to unwind nonspecific DNA, we performed a fluorescence-based helicase assay. Somewhat surprisingly, all mutants except the control K340H mutant exhibited similar helicase activity on a heteroduplex nonspecific DNA substrate (Fig. 5). These results suggest that under these experimental conditions Rep68 can unwind DNA even in the absence of large complexes or, alternatively, that an oligomeric complex that is stabilized by a different interface is necessary for Rep-mediated DNA unwinding. Strand- and site-specific nicking activity, however, appeared to diminish strongly when oligomerization was disrupted in Rep68. As expected, the oligomerization-deficient Rep mutants that failed to bind specific DNA also failed to nick supercoiled plasmid DNA containing RBS and *trs* sequences (Fig. 6A and B). The Rep68*-Y224F mutant, despite retaining the ability to bind specific DNA substrates, showed only some residual nicking activity.

### Rep oligomerization is important for transcriptional regulation of AAV genes.

**FIG 7:**
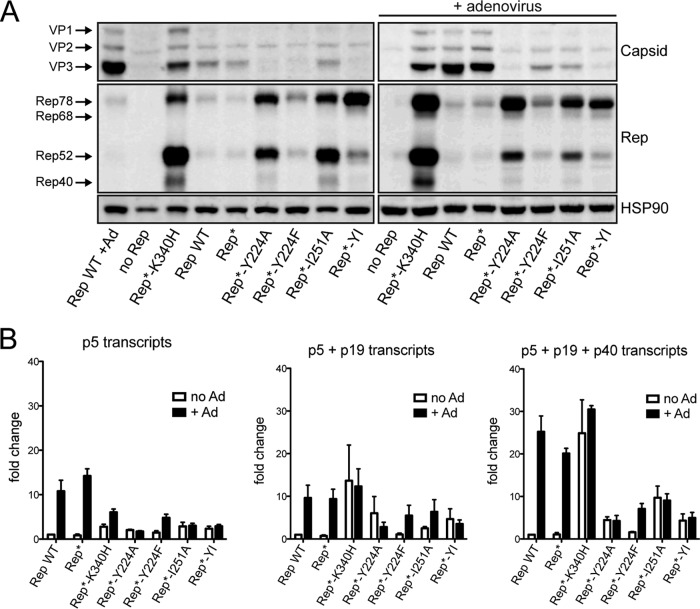
Rep oligomerization is important for transcriptional regulation of AAV genes. (A) Western blot showing Rep and Cap protein levels under conditions permissive (+ adenovirus) and nonpermissive for AAV replication. The first lane in the left panel is equivalent to the third lane in the right panel. (B) The transcription levels of AAV genes were analyzed under the same conditions shown in panel A. RNA levels were measured by RT-qPCR using three primer-probe mixes detecting RNA from the p5 promoter only, from p5+p19, and from p5+p19+p40. The fold change was calculated relative to the mRNA levels in the presence of WT Rep but in the absence of adenovirus. Data are from three independent experiments and are represented as the mean ± SEM.

In addition to their role in AAV DNA replication, the Rep proteins coordinate the temporal regulation of transcription of the viral genome during the AAV life cycle. In the absence of helper virus, the Rep proteins participate in repressing transcription from the three viral promoters, p5, p19, and p40, ensuring minute levels of expression of the viral proteins. In the presence of helper virus, i.e., during a productive infection, repression of the p5 promoter is lifted by the adenoviral E1A protein (53), and binding of Rep to the p5 promoter or the ITRs leads to transactivation of the p19 and p40 promoters (54–56). The p5 promoter itself is also controlled by Rep, which can act both as a repressor and as an activator through binding at the p5 or ITR RBS, respectively (56, 57). The net result is a self-regulatory loop that generates protein levels that are tightly controlled and are optimal for AAV replication and packaging (58). Two mechanisms of Rep-mediated repression have been identified: direct repression through binding at the RBS in the p5 promoter and indirect repression that requires the ATPase activity of Rep (19). In light of the dependence of transcriptional regulation on Rep binding to the p5 promoter and ITR, we assessed whether the oligomerization mutants also displayed defects in transcriptional activity resulting in altered protein expression levels. As a control, we again used the K340H ATPase mutant, which has been shown to lead to the expression of exceedingly high Rep protein levels under conditions permissive for AAV replication (48). Cells were transfected with the various AAV infectious plasmids, and Rep and Cap protein levels were determined in the presence and absence of adenovirus coinfection. In the presence of WT Rep and the absence of adenovirus, we expected low levels of both Rep and Cap proteins. In the presence of adenovirus, Rep protein levels peaked at about 30 h after infection and then slowly decreased, while Cap levels increased with viral DNA replication (56). Because we harvested the cells at 72 h posttransfection, we expected to see only slightly higher levels of the Rep proteins but significantly higher Cap expression levels compared to those in cells that were not coinfected with adenovirus. As shown in Fig. 7A, we observed strikingly high Rep protein levels in cells transfected with the mutated Rep proteins, including the control K340H mutant. Once more, we observed that the Y224F mutant showed an intermediate phenotype represented by a very modest increase in Rep protein expression levels (Fig. 7A). The Cap protein levels, on the other hand, were found to be lower in cells expressing the mutant proteins; we detected high Cap levels only in the presence of Rep proteins that support the AAV life cycle, with the sole exception being the K340H mutant (Fig. 7A). The same trend was observed both in the presence and in the absence of adenovirus infection, although the Cap levels were significantly lower in the absence of helper virus. These results suggest that the oligomerization mutants failed to regulate the expression levels of the AAV proteins, most likely by failing to autoregulate the p5 promoter through RBS binding. In view of these important differences in protein amounts, we assessed the levels of AAV transcripts by RT-qPCR (Fig. 6C). Because all AAV RNAs use the same polyadenylation signal, we were not able to quantify the p19 and p40 transcripts separately from the p5 transcripts. As expected, with all primer sets used, which targeted p5, p5+p19, and p5+p19+p40, we observed an increase in mRNA levels in response to adenovirus coinfection in the presence of Rep proteins that support AAV replication. In the presence of the Y224F mutant, the response to adenovirus was still present but was nevertheless reduced compared to that in the presence of WT Rep. The oligomerization-deficient Y224, I251, and Y224-I251 mutants, which were unable to bind the RBS at the p5 promoter, had higher basal mRNA levels, varying between 2- and 10-fold compared with those of the WT, and did not respond to adenovirus infection. In the context of adenovirus coinfection, however, the differences in mRNA levels did not correlate with those observed for the protein levels, suggesting that changes in posttranscriptional regulation also contribute to the altered protein expression levels. Rep-mediated posttranscriptional regulation has been observed before, but its mechanism remains unknown (59). The K340H mutant, which oligomerized but failed to support AAV replication, had considerably higher basal mRNA levels than WT Rep, possibly explaining the very high Rep and Cap protein amounts observed, and the presence of adenovirus did not lead to a clear change in mRNA levels. Taken together, our data support a model in which Rep oligomerization is important for the gene regulatory function of Rep, potentially through p5 RBS binding, which is necessary to achieve an appropriate transcription profile.

## DISCUSSION

The limited genome capacity of small viruses, such as adeno-associated virus, has driven the evolution of highly multifunctional nonstructural proteins that combine several enzymatic functions necessary to support the viral life cycle. In AAV, the Rep proteins are responsible for orchestrating the entire viral life cycle, from transcriptional regulation to replication and packaging as well as Rep-mediated integration. The combination of several enzymatic functions, including DNA binding, nicking, and unwinding, and the ability to interact with a multitude of DNA substrates and proteins allow the Rep proteins to support replication. However, the coordination of all these functions would require a tightly controlled system, which we envision could be provided by the different oligomeric states that the protein assumes. During the AAV life cycle, Rep has to catalyze reactions on different DNA substrates, including initiation of DNA replication, recognition and nicking of the *trs*, and binding to the p5 promoter, in order to provide transcriptional regulation. It has been shown that Rep can form different oligomeric species *in vitro* both in the absence of DNA and in the presence of different DNA substrates (24, 25), allowing an additional layer of regulation of the Rep activities during the AAV life cycle. To fully understand the mechanism of action of Rep on its different substrates, it is essential to identify the oligomeric complexes formed with the different DNA molecules. For example, it has been shown that Rep68 forms a double octameric ring in the presence of single-stranded DNA as well as on forked helicase substrates (24), whereas other reports have suggested that Rep68 forms hexamers when it is bound to double-stranded DNA (50, 60). However, the importance of these complexes for the viral life cycle has not been formally addressed, and while it is clear that Rep oligomerization is functionally relevant, data on possible oligomerization interfaces remain scarce. Smith and colleagues identified two regions—residues 151 to 188 and residues 334 to 347—that, when deleted, disrupt Rep oligomerization; however, they did not investigate the functional consequences of these deletions (50). Intriguingly, residues 334 to 346 include the ATP binding site that has been shown to be part of the oligomeric interface in PV E1 and SV40 LTag hexamers. A more recent report showed that one residue, R107, which was initially identified for its role in integration, origin binding, and nicking and which was shown to be in direct contact with origin DNA, is also essential for oligomerization (24, 61). Finally, we and others have shown that the interdomain linker of Rep78/68 and, in particular, the Y224 residue are critical for Rep oligomerization (28, 32).

Building on our previous studies, we further characterized the role of residue Y224 in Rep oligomerization. Replacement of Y224 with residues with different properties had various consequences on Rep68* oligomerization. More specifically, replacement of Y224 with small hydrophobic residues severely impaired oligomerization, while the Y224F mutant with the more conservative mutation retained the ability to oligomerize (Fig. 1), suggesting that Y224 participates in the formation and the stabilization of a hydrophobic interface. This hypothesis was supported by a model of a dimeric Rep-Rep interaction built from the pseudodimer observed in the crystal structure of Rep40 using an extended Rep40 molecule (33). In this model, a large interface that resembled the interface formed by the PV E1 protein and included residues from the Walker A and Walker B motifs, PS1βH, and the β2β3 loop was formed (Fig. 2). Furthermore, all the helices in the OD also participated in the interface and formed a hydrophobic pocket, emphasizing the relevance of this subdomain in Rep oligomerization. More specifically, linker residue Y224 on the extended α-helix 1 of one Rep molecule interacted with residue I251 and with the main chain carbonyl oxygen of residue N254 on α-helix 3 of the other Rep molecule (Fig. 2C). Mutating I251 to alanine in Rep68 alone or in combination with Y224A confirmed that this residue is important for Rep68 oligomerization. Importantly, because none of the residues located in the OD that we identified to be participants in the oligomeric interface are part of the catalytic sites described within the Rep proteins, the consequences of these mutations on the functions of the Rep proteins are likely to be due to oligomerization defects. We showed that the oligomerization-deficient Y224A, I251A, and Y224A-I251A mutants were unable to replicate AAV DNA and failed to support the production of recombinant AAV. Our data suggest that these defects are caused by the loss of DNA binding and origin nicking activities by the mutant Rep proteins and confirm that Rep oligomerization is critical for its function in support of the AAV life cycle. We also assessed the consequences of a more conservative mutation, Y224F, on Rep function. This substitution maintained the bulky aromatic character of the residue, a feature that is conserved in the OD of SF3 helicases and other related proteins (28). The Rep68*-Y224F mutant retained the ability to oligomerize but formed the large 13S complexes less efficiently than Rep68* did (Fig. 1). Interestingly, the binding of RBS-containing DNA did not appear to be compromised by this mutation. The Rep68*-Y224F nicking activity, on the other hand, was severely impaired, possibly explaining the low levels of viral replication observed (Fig. 6). In view of the oligomeric behavior of the Y224F mutant, these results suggest that this mutant retains the ability to form an oligomeric complex sufficient for RBS-mediated DNA binding but fails to promote the subsequent DNA nicking step. How this transition is affected, however, is not clear. One intriguing possibility is that the initial Rep binding to the RBS and melting of the origin promote the recruitment of further Rep78/68 molecules and the assembly of a second, larger Rep-DNA complex that is necessary for the nicking reaction. Residue Y224 and, more generally, the OD could help stabilize the formation of this complex, allowing a shift in the interaction with the origin DNA to allow the *trs* nicking reaction to take place. The Y224F mutation was previously identified in a study by Walker et al. to be important for Rep function (62). In contrast to our findings, however, those authors reported that the Y224F mutant was deficient in ITR binding, endonuclease, DNA helicase, and ATPase activities. The cause of this difference may be due to a different experimental strategy or could possibly be explained by the presence of a maltose-binding protein (MBP) tag (62), which may affect the already weakened oligomerization potential of Rep68-Y224F.

SF3 helicases are thought to function as oligomeric complexes, as is the case for PV E1 and SV40 LTag, which form active hexameric complexes. Surprisingly, all the oligomerization-deficient mutants described here were still able to unwind a heteroduplex DNA substrate (Fig. 5). This suggests that interaction with and unwinding of 3′-tailed substrates do not require the formation of large Rep oligomers, consistent with the helicase activity of Rep40, or, alternatively, that the presence of heteroduplex DNA and ATP stabilizes the formation of an oligomeric complex independently from the oligomeric interface described here. Rep40 is monomeric in solution, forms transient dimers in the presence of ATP, and retains helicase activity, albeit at a level lower than that observed with the large Rep proteins (28, 63). Therefore, although the mutants presented here do not form a complex with the *AAVS1* site, they could form transient oligomers in the presence of ATP that are able to unwind DNA. In our previous report, we introduced the possibility that AAV Rep proteins have evolved two distinct helicase modes (28): one that parallels the helicase activity of other SF3 helicases, requires oligomeric rings, and is performed by the large Rep proteins, and one that requires only a transient dimerization and that is characteristic for the activity of the small Rep isoforms. Thus, it is plausible that the mutants described here are still able to unwind DNA through the same mechanism used by Rep52/40, but they would not support the unwinding of a substrate that requires the helicase activity from oligomeric rings. On the basis of the different functions of the large and small Rep proteins in the AAV life cycle, it is tempting to suggest that melting of the AAV origin, which is mediated by the large Rep proteins, requires the formation of a stable oligomer that unwinds DNA by a mechanism analogous to that described for other SF3 helicases, while packaging AAV genomes into the viral capsids, which is efficiently carried out by the small Rep proteins, may proceed through a different helicase mode.

In addition to their role in supporting AAV DNA replication, the enzymatic activities of the Rep proteins are also essential for the correct transcriptional regulation of viral and cellular transcripts. Because it is known that the levels of Rep proteins are tightly regulated and not simply maximized to achieve efficient AAV DNA replication (58, 64), we assessed whether the expression levels of AAV proteins were affected by the oligomerization mutants. The K340H Rep mutant has been shown to fail in appropriately regulating the expression of the AAV genes, suggesting that the ATPase/helicase activity of Rep is involved in transcriptional regulation (48). A different mechanism of transcriptional repression that is dependent on the RBS binding activity of Rep78/68 has also been demonstrated (20). Thus, two mechanisms of repression—one that is RBS binding dependent and one that is helicase domain dependent—exist and likely act in concert to precisely regulate the levels of expression of the Rep proteins. In this study, we show that regulation of AAV gene expression is impaired in the presence of Rep oligomerization mutants that do not bind p5 or *AAVS1* DNA, indicating that at least one of two mechanisms of repression is impaired. Our results suggest that oligomerization of the large Rep proteins is necessary for the correct regulation of the transcription of all AAV promoters (Fig. 7). More specifically, the oligomerization-deficient mutants fail to induce transcription of the viral promoters upon infection with the helper virus adenovirus, and, in addition, both large and small Rep protein levels increase substantially in the presence of oligomerization mutants. The presence of the Y224A mutation creates a Kozak sequence at the p19 promoter stronger than that achieved with WT Rep68, but this is not sufficient to explain the differences in protein levels observed, in particular for Rep78. In addition, the increase in Rep52 expression in the presence of the Y224A-I251A mutations is modest compared to that observed in the presence of Y224A alone. The differences in protein levels observed in the presence of adenovirus, however, cannot be explained by the RNA levels alone, suggesting that there is some level of posttranscriptional control that may also be Rep dependent. A function for AAV Rep in this context was previously suggested in a study by Trempe and Carter, where it was observed that the regulation of gene expression at a transcriptional level alone was not sufficient to explain differences in protein levels (59). Our data also support a role for an oligomeric complex of Rep in regulating protein levels posttranscriptionally. Understanding the mechanism behind this potential uncharacterized function of AAV Rep proteins may reveal a new layer of complexity in the role that the Rep proteins play in coordinating the AAV life cycle.

In conclusion, our study identifies and describes an essential Rep-Rep protein interface that is involved in the formation of Rep complexes and demonstrates its functional relevance throughout the AAV life cycle. Our study focuses on residues that are part of the α-helical bundle located upstream of the helicase domain and strengthens the suggestion that this subdomain of Rep plays a role as a bona fide oligomerization domain. The identification of the oligomeric interfaces of AAV Rep like the one described here and further structural and functional characterization of Rep oligomeric complexes, particularly in the presence of different DNA substrates, will provide additional insights into the molecular mechanisms of Rep-mediated transcriptional regulation and AAV DNA replication, as well as Rep-mediated integration.
